# Leveraging Urban Water Distribution Systems with Smart Sensors for Sustainable Cities

**DOI:** 10.3390/s24227223

**Published:** 2024-11-12

**Authors:** Anaraida García Baigorri, Raúl Parada, Victor Monzon Baeza, Carlos Monzo

**Affiliations:** 1 Faculty of Computer Science, Multimedia and Telecommunications, Universitat Oberta de Catalunya (UOC), Rambla del Poblenou 156, 08018 Barcelona, Spain; agarciabai@uoc.edu (A.G.B.); vmonzon@uoc.edu (V.M.B.); cmonzo@uoc.edu (C.M.); 2 Centre Tecnològic de Telecomunicacions de Catalunya (CTTC), Av. Carl Friedrich Gauss, 7-Edifici B4, 08860 Castelldefels, Spain

**Keywords:** natural environmental software, water distribution systems, optimization, sensoring, energy efficiency

## Abstract

Optimizing urban water distribution systems is essential for reducing economic losses, minimizing water wastage, and addressing resource access gaps, particularly in drought-prone regions impacted by climate change. We apply advanced artificial intelligence (AI) techniques and the Internet of Things (IoT) to optimize water networks in Spain using simulation. By employing EPANET for hydraulic modeling and a linear regression-based algorithm for optimization, we achieved up to 96.62% system efficiency with a mean absolute error of 0.049. Our approach demonstrates the potential to conserve up to 648,000 L of water daily at high-demand nodes, contributing to substantial resource savings across urban water networks. We propose a global architecture utilizing Low Power Wide Area Network and Low Earth Orbit solutions for widespread deployment. This study underscores the potential of AI in water network optimization and suggests future research avenues for implementing the proposed architecture in real urban water systems.

## 1. Introduction

Water is vital for urban life, supporting drinking, sanitation, agriculture, and industry. Population growth, climate change, and inefficient management have led to shortages, threatening health and economic stability, especially in drought-prone areas like Spain. Efficient water management is essential for reducing waste and ensuring equitable access. This study aims to optimize urban water distribution systems in Spain by applying artificial intelligence (AI) and the Internet of Things (IoT) to minimize economic losses and resource gaps. The IoT paradigm significantly enhances water distribution systems by enabling real-time monitoring, sensor-driven data collection, and seamless communication between system components. This allows for predictive maintenance, identification of leaks, and improved efficiency by dynamically adjusting water flow based on demand patterns. In [1], a *National Hydrological Plan* alongside *Basin Hydrological Plans* and an *Agrarian Irrigation Plan* were proposed to effectively manage water resources, promote efficient use, and safeguard ecosystems to combat climate change and water resource anomalies. In [2], Spain’s geographic position was noted as making it vulnerable to droughts due to its subtropical climate, risking drinking water supplies. The study highlighted increasing demands leading to a structural deficit in the southeastern Iberian Peninsula, inefficiency in storage systems, and limited knowledge of water losses. Martínez Gil [3] emphasized the need for a change in water management, proposing the *New Water Culture*, which advocates for a holistic understanding of water management based on scientific reasoning, the precautionary principle, and the fraternity between humans and nature.

Since these early studies, the need for more efficient water infrastructure management has become evident, with studies highlighting an imminent future of water scarcity. Proposed solutions often did not rely on advanced technology due to its immaturity at the time. However, some technological solutions were suggested, such as using geographic information technologies like remote sensing to improve water planning and management in irrigation communities [4]. The OPTIMA project focused on efficient resource management based on the EU Water Framework Directive, using a client-server approach and optimization algorithms [5]. Other proposals included forecasting reservoir water levels [6,7]. Our research contributes directly to the United Nations Sustainable Development Goals (SDGs) [8], particularly SDG 6: Clean Water and Sanitation, and SDG 11: Sustainable Cities and Communities. By optimizing urban water distribution, our work promotes efficient water management, which is essential for ensuring clean and sustainable water access in urban areas. Addressing SDG 6 aligns with our goal of minimizing water wastage and ensuring equitable access to this critical resource, especially in drought-prone regions. Additionally, this study supports SDG 11 by enhancing urban resilience and infrastructure sustainability. Our proposed architecture, which integrates IoT and AI, can be adapted to varying urban settings and can inform public policies on resource management and smart city planning, reinforcing sustainable urbanization efforts. The objectives of this study are (1) to analyze diverse urban areas in Spain, focusing on varied climatic conditions related to real-world use cases, given the country’s climate crisis concerning water reserves; (2) to aim to create and simulate urban water networks using an open-source hydraulic simulator such as EPANET software [9] in various Spanish cities of reference; (3) optimizing their infrastructures with EPANET, which will support future comparative and evolutionary projects in Smart Cities and provide a framework for future research; and (4) to justify the optimization of urban water distribution systems (WDSs) to save resources daily, thereby contributing to more efficient and sustainable urban management. The rest of this paper is structured as follows: Section 2 reviews related work on WDSs. Section 3 describes the tools used. The methodology applied is shown in Section 4. The simulation results are presented and discussed in Section 5, including a proposed architecture for real-world deployment. Finally, Section 6 summarizes the findings and suggests future research directions.

## 2. Related Work

Several studies explore optimizing water networks using simulations and artificial intelligence (AI) algorithms. Ref. [10] applies EPANET for designing WDS in Jharkhand, focusing on both urban and rural management. Ref. [11] develops a management system for water resources with wireless sensors and a mobile app. Ref. [12] creates a framework with EPANET version 2.0. and MATLAB R2024b for optimal disinfectant dosage in intermittent water supplies. Ref. [13] presents a methodology for the efficient modeling of water distribution networks (WDNs) using EPANET in a preliminary version, aiming to reduce computational load. Ref. [14] uses EPANET 2.0 to simulate water quality in pipelines and assess interventions. Ref. [15] employs EPANET in a digital twin model for comprehensive WDN analysis. Ref. [16] optimizes roughness coefficients with a Genetic Algorithm (GA) but does not focus on water node optimization. Ref. [17] optimizes water quality parameters in WDSs using EPANET. Ref. [18] creates an EPANET-based model for Kuwaiti water networks, evaluating water quality and age. Ref. [19] introduces the Fuzzy C-means Adaptive Differential Evolution (FCADE) algorithm for WDN challenges integrated with EPANET. Ref. [20] presents a mobile app using the Arduino MCU platform for leak detection and pressure measurement. In contrast, our study simulates and optimizes nodes in urban water networks to reduce wastage. Ref. [21] explores the use of LEO satellites for remote water level monitoring and optimizing energy consumption. Ref. [22] integrates the IoT and Blockchain for agricultural water management. Ref. [23] reviews AI applications in water conservation, covering system components and their interdependencies. Ref. [24] designs a solution for renewing critical water infrastructure using Industry 4.0 technologies for Smart Cities. Ref. [25] discuss the development of a web-based EPANET simulation and visualization for large water networks to support water management. In [26], a water monitoring system based on pH measurements has been introduced to modernize urban water distribution systems. However, unlike as proposed here, this system does not demonstrate application in real urban networks. The approach in [26] employs traditional predictive methods and uses a NODE MCU sensor architecture. Recently, [27] presented a series of theoretical approaches for transforming urban water distribution systems. This proposal evaluated technologies and predictive models statistically, focusing on theoretical optimization rather than practical application. Table 1 compares these works with our study, focusing on aspects such as water analysis (study of real water networks), AI (mention or implementation of cognitive algorithms), optimization [Opt.] (improving the performance of current infrastructures), technology [Tech.] (use of communication mechanism, sustainability [Sust.] (reducing carbon footprint and water wasting), and architecture [Arch.] (proposing real designs).

As can be seen in Table 1, our work covers most of the aspects found in the literature. We aim not only to leverage an open-source simulator to highlight the critical situation of current reservoirs, but also to optimize the nodes and propose a novel architecture to be deployed on urban water networks. Therefore, we utilize EPANET to model an urban water network and Python 3.9 to develop optimization algorithms.

## 3. Environment and Simulation Scenario

**Figure 1:**
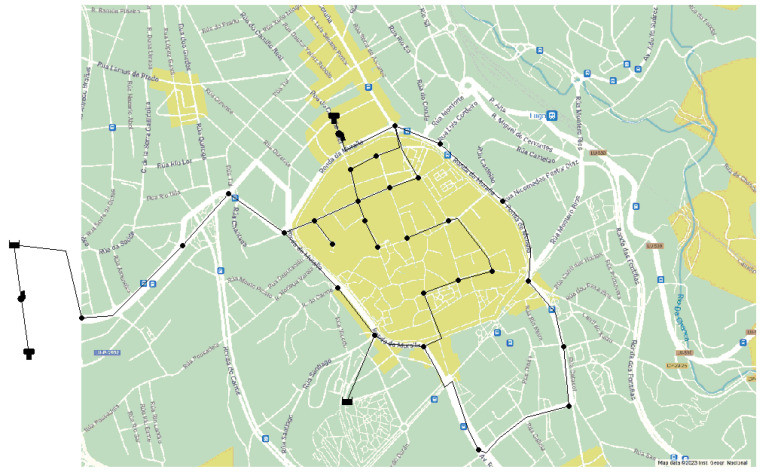
Node deployment in Lugo.

**Figure 2:**
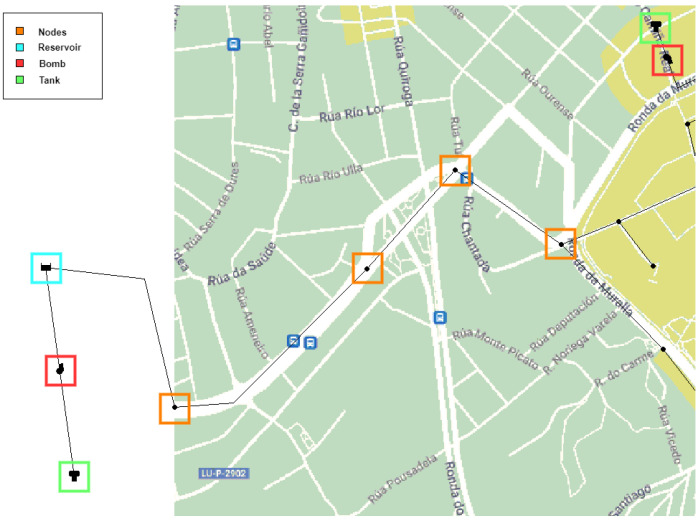
Network modeling symbol representation in Lugo.

**Figure 3:**
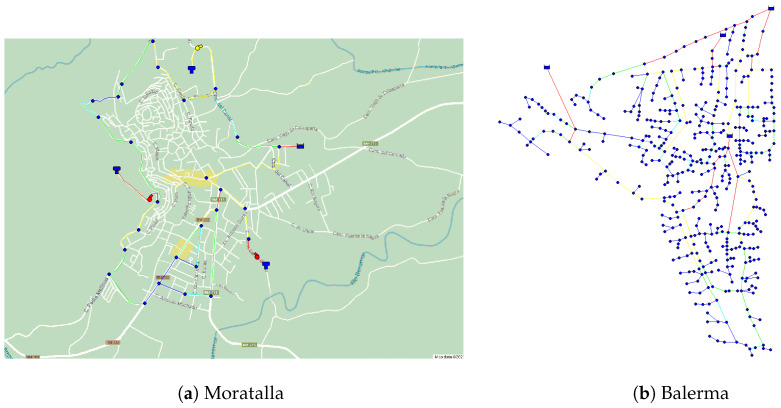
Node deployment in Moratalla (**a**) and Balerma (**b**).

EPANET [9], an open-source hydraulic modeling software, is widely used for designing and analyzing potable WDSs. It employs a node and link approach to represent the water distribution components. Our study used templates of selected areas from Google Maps and Google Earth to model networks. These templates preserve the original location and shape, providing realistic deployments. The map images were exported to the bitmap format required by EPANET. We selected three diverse urban areas in Spain for our simulations: Lugo, Moratalla, and Balerma. It was determined that including at least one scenario with fewer than 30 nodes would allow the exploration of the relationship between resource optimization and node numbers. In practice, we selected two scenarios with fewer than 30 nodes and one with more than 30 nodes. Despite the similar node counts in the two former scenarios, differences in height above sea level and population allow for diverse results without needing additional nodes for this research. The scenarios are as follows. (1) Lugo is characterized by short, dry, hot summers and long, cold winters with heavy precipitation. Annual temperatures range from 2 °C to 27 °C, occasionally reaching −3 °C or 33 °C and, 1017 mm of precipitation annually (mean). Lugo is an urban city in northwest Spain with a population of 98,189 inhabitants, an altitude above sea level of 465 m, and the average water consumption per inhabitant is 130 L/day. These characteristics allow us to obtain the specific base demand for this area, which is 147.73 L/s. A margin of 15% of the base demand can also be considered, allowing it to fluctuate between 125.57 ≤ 147.73 ≤ 169.88 L/s. A total of 28 nodes, 2 reservoirs, 2 tanks, 31 pipelines, and 2 pumps are deployed for Lugo in EPANET. Figure 1 shows the location of each node. This distribution has been designed to cover the urban core and simulate values existing in the downtown area, which has the highest number of dwellings. Figure 2 displays in detail how each of the above elements is represented in the EPANET software, where nodes are highlighted in orange, reservoirs in turquoise, pumps in red, and finally, tanks in green. (2) Moratalla is an urban city in southeastern Spain with a population of 7753, an altitude of 681 m above sea level, and an average water consumption of 180 L/day per inhabitant, leading to a base demand of 16.15 L/s. It experiences warm, humid summers and long, cold, partly cloudy winters, remaining dry year-round. Temperatures range from 4 °C to 33 °C, occasionally dropping below 0 °C or exceeding 36 °C, and 455 mm of precipitation annually (mean). This city’s water network includes 27 nodes, a reservoir, 3 tanks, 30 pipes, and 3 pumps. The layout of these elements is shown in Figure 3a. Unlike Lugo, Moratalla’s area was modeled to simulate consumption values and achieve a broader range of study results. (3) Balerma is an urban area in Almeria, southeastern Spain, with a population of 4779, an altitude of 3 m above sea level, and an average water consumption of 100 L/day per inhabitant, resulting in a base demand of 5.53 L/s. It has a warm climate year-round, with an average annual temperature of 19 °C and 195 mm of precipitation annually (mean). The WDN in Balerma, based on a study by Bi et al. [28], includes 443 nodes, 4 reservoirs, and 454 pipes. This scenario involves complete area modeling, providing a population simulation. The network layout is shown in Figure 3b.

These three areas represent a range of climatic and geographical conditions, from oceanic and mountainous to Mediterranean and flat landscapes.

Python is primarily used for data processing within the machine learning framework. It plays a crucial role in implementing linear regression (LR) models using data generated from the EPANET simulations. Two algorithms utilizing LR are proposed; one compares the base demand with node demand, while the other compares the base demand with pipe diameter. LR has been selected due to its low-computational complexity, only O(n * p^2^) being *n* the number of data points and *p* the number of features, in contrast to O(n^2^) from GA approaches used in the related work. Hence, in taking this approach, we contribute to a more sustainable solution.

## 4. Proposed Methodology

**Figure 4:**
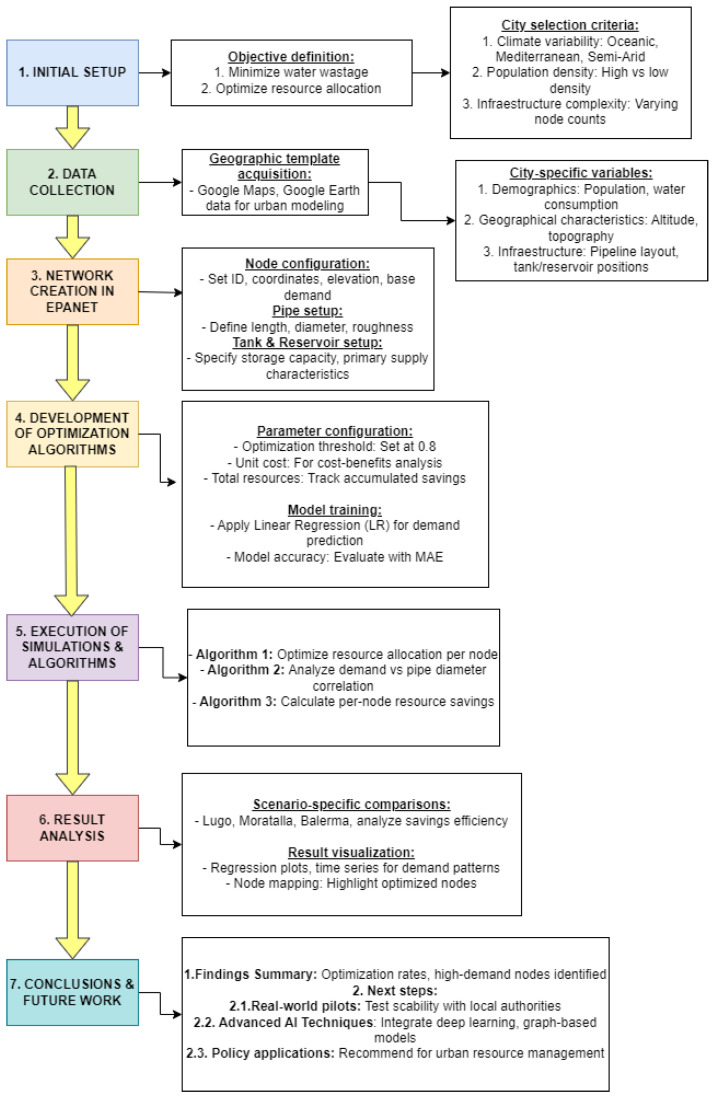
Flowchart Methodology.

The methodology adopted for this study comprises seven stages, as illustrated in Figure 4. In this section, we specify the data collection and project initiation phases, along with the creation of the network using the EPANET tool, justifying its selection and outlining the steps related to the algorithms. The subsequent sections will detail the results and conclusions derived from this approach.

### 4.1. Networks Creation Using EPANET

We used EPANET to simulate hydraulic systems, including nodes, pipes, tanks, and reservoirs. Nodes represent connection points with properties like ID, coordinates, elevation, and base demand. Pipes connect nodes and their key properties are length, diameter, and roughness. Tanks store water with modifiable properties such as elevation and capacity. Reservoirs are primary water supply sources.

City selection was based on diverse criteria, including climate, population density, and infrastructure complexity. We selected cities with distinct climate zones (oceanic, Mediterranean, and semi-arid in the case of Lugo, Moratalla, and Balerma, respectively) to ensure our approach’s applicability to various environmental conditions. Additionally, factors such as altitude, average annual rainfall, and urban infrastructure were considered to observe how these variables impact water distribution optimization. This diversity enables our models to address a range of urban water challenges, enhancing generalizability.

### 4.2. Optimization Algorithm

Three algorithms were developed to optimize the above-created WDNs. We use an LR technique to feed our simulation output. When programming LR, three global variables or parameters have been considered:

1. threshold_optimization: This indicates when a node has successfully optimized its water demand resources. It is set initially to 0.8, meaning optimization applies once the threshold is below this value. It should not exceed 1.0 (100%). An 80% threshold is used here, meaning optimization occurs when lps_demand ($L_{D}$) is less than 80% of base_demand ($B_{D}$). Thus, $L_{D}$ values must be at least 20% lower than $B_{D}$, reflecting theoretical demand based on area characteristics like average water consumption per inhabitant and population.
2. Unit_cost: This parameter calculates the cost per unit of base demand, enabling the estimation of total resource costs. It is critical for the evaluation of the financial savings achieved through water optimization. It has no impact on the LR; however, it is used to calculate the costs when predicting with the model, which will allow the estimation of the total resource costs in the network, serving as a cost analysis. For this situation, it has been chosen to be set initially to 0.5, a random value less or equal to the unit. The user can set their own value. The difference concerning the initial value is that the unit cost will be reduced proportionally to the value set, with no interference from the variation in unit_cost in the LR, as demonstrated by tests carried out varying this parameter from 0.25 and 0.75. That parameter has been added to obtain details on the number of resources that could be saved when the optimization condition is reached at nodes.
3. total_resources: This parameter is set initially to 0 because that symbolizes the starting point where total resources will start to accumulate. This is the ideal starting point, as with each LR performance, total resources that will have been optimized will be obtained; meanwhile, if the value is initialized to a state other than 0, it will always start from a state that is not the one we want for our analysis. It should be pointed out that this parameter is related to the unit_cost parameter, so any change in its value has a proportional effect upon unit_cost. This parameter can collect the total resources used in the system to perform node optimization.

Next, we describe the three above-mentioned algorithms. Algorithm 1 has the functionality to read the provided dataset, which in this case contains the base demands and the lps (liter per second) demands of the nodes of our water distribution scenarios. A machine learning model is trained to obtain a prediction of the $L_{D}$ based on the $B_{D}$, where the model’s performance will be evaluated through the mean absolute error (MAE). Subsequently, a loop is performed at each node, where the cost of the resources that would have to be used to supply that demand will be calculated. For example, suppose the simulated demand, being the $L_{D}$ value, is lower than the $B_{D}$ at the threshold determined (80%). In that case, a message on the screen will indicate that optimization has been achieved, reducing the resources by 10%. The 10% reduction criterion was selected based on our preliminary simulation results. When LD (Local Demand) is lower than BD (Base Demand), a conservative reduction of 10% ensures sustainability without risking undersupply, particularly when the system operates near the 80% threshold, a critical level in balancing resource conservation and user demand. In the opposite case, a message will be displayed indicating that optimization has not been achieved, where the total resources of all nodes will be accumulated. This algorithm optimizes water distribution using an ML model to predict when the $L_{D}$ will be less than the $B_{D}$ at the threshold to be determined, reducing the resources supplied to those nodes.

Global variables are defined, and then the dataframe is loaded, consisting of $B_{D}$ and $L_{D}$, extracted from the EPANET simulation. In lines 4 to 6, the model is trained and evaluated by training the LR model, predicting values, and calculating the MAE error. For the optimization process, a dataframe is loaded (line 8) with data from each node, where for each node, the optimized resources will be calculated, where the id, base demand, and lps demand will be obtained, the initial resources will be calculated, and if the $L_{D}$ is below the threshold, a 10% reduction in resources will be applied; otherwise, it will not be considered to have been optimized, and the resources that are optimized will be accumulated. The aim of Algorithm 1 is to predict the lps demand to optimize the resources by comparing the actual demand with the predicted demand at each node, where the final purpose is to reach water resource reduction through optimization of water demand.
**Algorithm 1** Water Distribution System Optimization 1:**Globals:** threshold_optimization=0.8, unit_cost=0.5, total_resources=0 2:**Load Data:** $X\leftarrow df[{[}^{\prime }base\_demand^{\prime }]]$, $y\leftarrow df[{[}^{\prime }lps\_demand^{\prime }]]$ 3:**Train Model and Evaluate:** 4:X_train,X_test,y_train,y_test← train_test_split(X, y, 0.2, 42) 5:model← LinearRegression().fit(X_train, y_train) 6:mae← mean_absolute_error(y_test, model.predict(X_test)) 7:**Optimization Process:** 8:df← pd.read_excel(’city_$L_{D}andB_{D}$.xlsx’, names=[’node_id’, ’base_demand’, ’lps_demand’]) 9:**for** index,row **in** df.iterrows() **do**10: $node\_id\leftarrow row{[}^{\prime }node\_id^{\prime }]$, $base\_demand,lps\_demand\leftarrow row[{[}^{\prime }base\_demand^{\prime }{,}^{\prime }lps\_demand^{\prime }]]$11: resources←base_demand×unit_cost12: **if** lps_demand<base_demand×threshold_optimization **then**13:  **print**(“Successful optimization at node”, node_id)14:  resources←resources×0.915: **else**16:  **print**(“Optimization was not achieved in the node”, node_id)17: **end if**18: total_resources←total_resources+resources19:**end for**

Algorithm 2 mainly implements a linear regression model to predict the base demand as a function of pipe diameter in a water distribution environment, where the data loaded into the model come from the results of the simulations, where the measurements of the selected pipe diameters and the base demand corresponding to each diameter are collected for all the nodes deployed in each scenario. Data arrays of diameter and base demand data are extracted from the dataframe, and a linear sklearn regression model is created to be trained with the diameter data (as the independent variable) and base demand (as the dependent variable). Diameter is expected to have the shape that the model expects. Finally, the regression and intersection coefficient of the trained regression line are plotted. This linear regression is intended to derive a mathematical model that captures a relation between pipe diameter and water demand, where this model allows for the prediction of the demand for new diameter values.

Algorithm 3 has the function of reading the set of data provided, which, in this case, will contain the values of node_id, base_demand, lps_demand, and node_inhabitants, to calculate and analyze the potential water savings per node, and achieve the total savings of the network, considering that water savings will only be calculated in those nodes that meet the optimization condition of Algorithm 1. In addition, this algorithm will allow the identification of nodes with a greater opportunity to optimize consumption. The dataframe is loaded in lines 1 and 2, where the values of base_demand and lps_demand, extracted from the EPANET simulation, and node_inhabitants will be obtained. Subsequently, in lines 3 to 15, a function is defined to calculate the savings per node. Before calculating savings, it is necessary to set an optimization condition (line 6), to obtain savings only for those nodes that optimize resources. Considering the previous condition is fulfilled, it is necessary to contrast base_demand with lps_demand to calculate savings. As a result of the variation between both fields, savings in liters per second are obtained, resulting in the lps_saving variable. In this scenario, we want to obtain the savings in liters per day, so using the conversion factor, the units are changed, obtaining variable lpd_saving, and subsequently, a savings value per inhabitant will be obtained. This function is applied to each row of the dataframe, where the column called saving_lpd containing the value of savings per inhabitant of each node is added. Finally, the total resources saved are obtained by adding up all the individual savings. To enhance visual analysis, a condition is set that the nodes are ordered from the greatest to the least in terms of resource savings. The objective of Algorithm 3 is to obtain the individual savings per inhabitant after optimization in a node and provide the total savings achieved due to optimizing all the nodes that make up the network.
**Algorithm 2** Linear Regression Model Training and Prediction 1:**Load Data:** 2:df← pd.read_excel(’city.xlsx’) 3:$diameter\leftarrow df{[}^{\prime }diameter^{\prime }].values$ 4:$base\_demand\leftarrow df{[}^{\prime }base\_demand^{\prime }].values$ 5:**Create Linear Regression Model:** 6:model← LinearRegression() 7:**Train the Model:** 8:model.fit(diameter.reshape(−1,1),base_demand) 9:**Extract Coefficients:**10:**print**(modelo.coef_)11:**Extract Intercept:**12:**print**(modelo.intercept_)13:**Generate Prediction Data:**14:line_diameters← np.linspace(min(diameter), max(diameter), 100)15:predictions← model.predict(diameters_line.reshape(−1,1))

**Algorithm 3** Water Savings Algorithm**Require:** Th: Threshold value for optimization
 1:**Load Data:** 2:df←ReadExcelfilenamedfile_name 3:**function** calculate_saving(row) 4: base_demand←Demandbasefromrow 5: lps_demand←Demandlpsfromrow 6: **if** demanda_lps<base_demand×TH **then** 7:  saving_lps←base_demand−lps_demand 8:  saving_lpd←saving_lps×60×60×24 9:  Inhabitants←Inhabitantsofnodefromrow10:  saving_inhabitant←saving_lpd/inhabitants11:  **return** saving_inhabitant12: **else**13:  **print** “Optimization not achieved in ”, $row{[}^{\prime }id\_node^{\prime }]$14:  **return** 015: **end if**16:**end function**17:**function** optimize_water_savings(df)18: $df{[}^{\prime }saving\_lpd^{\prime }]\leftarrow \mathrm{Apply}$ calculate_saving function to each row19: total_saving←Sumofsavingswheresaving_lpd>020: **print** “Total savings: ”, total_saving, “ liters/day”21: df_optimized←Selectrowswheresaving_lpd>022: df_optimized←Sortrowsbysaving_lpdindesc.ord.23: **return** df_optimized, total_saving24:**end function**

In terms of complexity, the algorithm used for optimizing water distribution networks with a linear regression model is efficient both in time and space. The time complexity of training the linear regression model is $O(n\times p^{2})$, where *n* represents the number of data points, and *p* represents the number of features. This is relatively efficient, especially when compared to genetic algorithm-based approaches common in related studies, which generally have a higher complexity of $O(n^{2})$. Additionally, the main optimization loop, which iterates over each node to calculate and apply resource-saving measures, has a linear time complexity of O(N), where *N* is the total number of nodes in the network. This ensures that the algorithm scales well with the number of nodes. The space complexity of the algorithm is also minimal. The linear regression model requires O(p) space to store model coefficients, which is manageable given that the number of features *p* is generally small compared to the number of data points *n*. Furthermore, the algorithm maintains memory for data storage and accumulations, resulting in an overall space complexity of O(N) to store node-specific information, with an additional O(1) space for constants such as the threshold_optimization, unit_cost, and total_resources parameters. Consequently, the total complexity of the algorithm is $O(n\times p^{2}+N)$ in time and O(N) in space, making it both time-efficient and space-efficient for real-time urban water network optimization.

The research compares the water distribution in three scenarios using EPANET and linear regression. Scenarios with fewer nodes relied on relief characteristics for diverse results. Algorithms developed aimed to optimize demand based on base demand and diameter through machine learning, achieving water savings at most nodes. Further analysis will explore these findings and their broader implications for sustainable water management.

## 5. Experimental Results

This section analyzes water distribution in three scenarios using EPANET and LR, focusing on base demand, flow optimization, and the relationship between demand and diameter. The number of inhabitants per node for each location is shown in Table 2, where the total number of nodes per location can also be seen. Those optimization criteria mentioned in the scenario description are highlighted.

The simulation from the described WDN (Section 3) provided data for the algorithms in Section 4.2, which integrated linear regression techniques. The regression line follows y=mx+n, where *m* indicates the slope (increase in base demand per unit diameter) and *n* denotes the Y-axis intersection (base demand when diameter = 0).

### 5.1. Lugo Scenario

A base demand of 147.73 L/s was calculated using:

(1) $$B_{D}=\frac{\text{average\_consumption}\;\times \;\mathrm{inhabitants}}{86,400}$$

where average consumption is 130 L/inhabitant-day and the population is 98,189. Negative flow values indicated opposite connection directions between nodes. Valves were simulated by adjusting pipe roughness. For a working point of 5 L/s, we obtained a diameter of 165 mm and a pump head of 32.5 mWc. The general pump characterization expression is:

(2) $$\mathrm{Head}=43.33-0.4334\times {\left(\mathrm{Flow}\right)}^{2}$$

**Figure 5:**
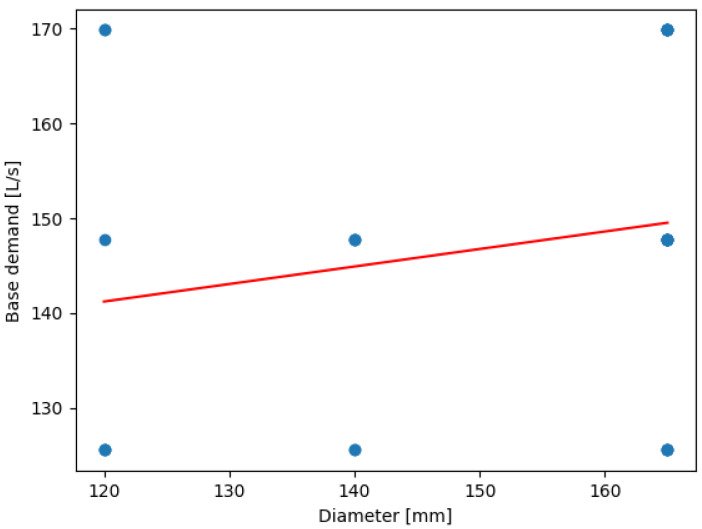
Linear regression between $B_{D}$ and pipe diameter in Lugo.

The node with the highest saving is the one with the ID “Junc Node28” with a total of 648,000 L/day, and the node with the lowest saving is the one with the ID “Junc Node8” with a total of 42,744.65 L/day. Total savings are the sum of all individual savings, resulting in 2,558,047.42 L/day. The linear regression in Figure 5 shows a positive trend, predicting node demand based on base demand values.

The linear regression between base demand and pipe diameter (Figure 5) indicates increased base demands with increasing pipe diameters.

(3) $$B_{D}=0.18472808\times \mathrm{diameter}+119.06156712$$

**Figure 6:**
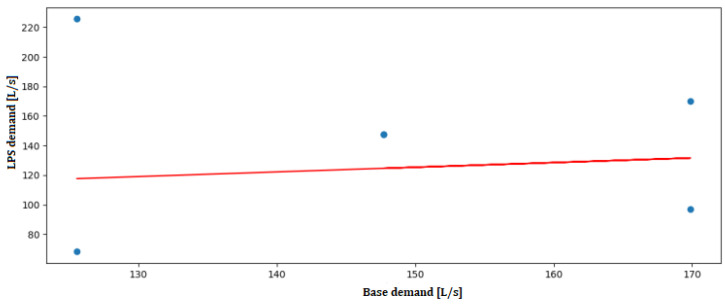
Linear regression between $B_{D}$ and $L_{D}$ in Lugo.

This positive trend reflects the expected increase in base demand as pipe diameters increase. Larger pipes are generally installed to handle greater water flows in areas of higher demand, such as those with denser populations or more commercial activity. The model’s slope (0.1847) indicates a moderate increase in base demand with diameter, suggesting a proportional scaling of resources required to maintain sufficient water supply across varying node demands. The high intercept value (119.06 L/s) is consistent with the baseline demands associated with the core network, which supports a large urban population. If we compare the values of base_demand and demand_lps obtained, we obtain the linear regression shown in Figure 6. This figure illustrates how the blue data points represent the data used to train the model: the simulation values. The red line represents the line of best fit generated by the model itself, which follows the trend of the data points. In this case, we select one of the base demand values previously calculated, such as 169.88 L/s to interpret the results. The model predicts that the node will demand nearly 140 L/s by drawing an invisible vertical line. If the calculated base demand is lower, the model predicts a lower demand from the node. The model thus determines the slope and intercept values that best predict the node’s demand.

**Figure 7:**
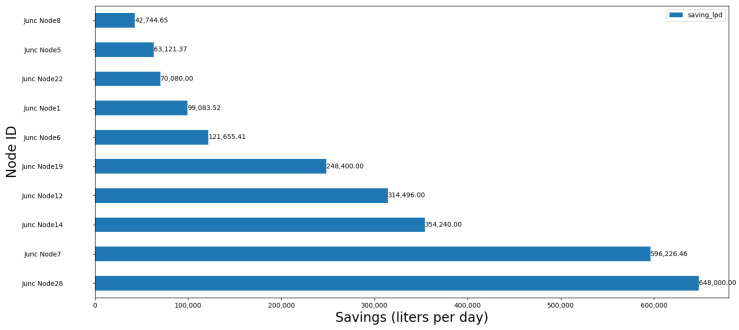
Resource savings per node optimized in Lugo.

The close fit between the predicted and actual values demonstrates the accuracy of the linear regression model in predicting local demand based on base demand values. The red trend line’s consistency with the data points further validates the model’s effectiveness in estimating demand changes across different nodes. The predicted values help to identify nodes where water supply may exceed actual demand, allowing for targeted optimization interventions. Figure 7 displays resource savings per optimized node, with the highest saving node achieving 648,000 L/day.

The variation in resource savings across nodes directly results from differing water demand patterns and network complexity within Lugo. For example, Node 28, with the highest savings, is located in a high-demand area, which likely serves a larger residential or commercial district. This node benefits the most from the optimization algorithm, achieving substantial water savings of 648,000 L/day. In contrast, Node 8, which sees far fewer savings (42,744.65 L/day), is likely located in a lower-demand zone, such as a peripheral residential area. These savings illustrate the importance of targeted optimization, where the algorithm can identify and prioritize nodes with the greatest potential for efficiency improvements. The highest-saving node (Node 28) achieves substantial water conservation, driven by its higher-than-average base demand, suggesting this node serves a high-demand area. Conversely, the lower savings at Node 8 reflect its smaller local demand, typical of less dense or peripheral urban areas. The cumulative savings across the system indicate substantial efficiency gains, further emphasizing the importance of targeted node optimization in maximizing water conservation.

### 5.2. Moratalla Scenario

**Figure 8:**
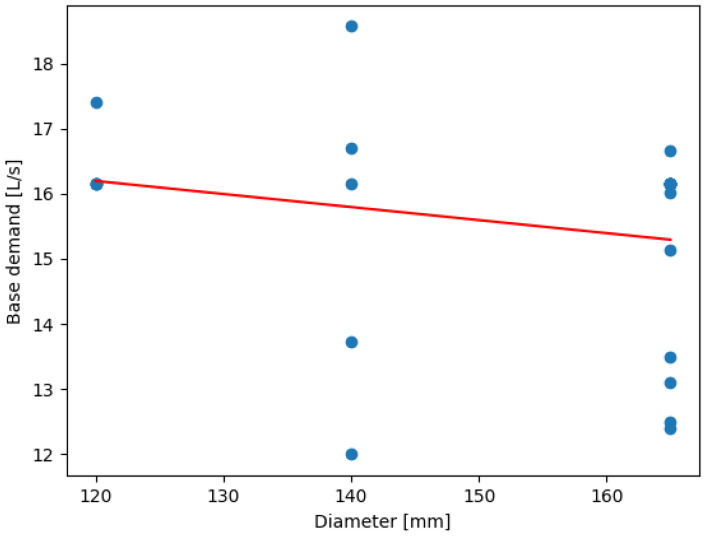
Linear regression between $B_{D}$ and pipe diameter in Moratalla.

Figure 8 shows the linear regression between base demand and pipe diameter for Moratalla. We can observe that the slope is negative, which results in lower base demands for larger pipe diameters. Transposing this condition to real conditions would suggest that larger diameter pipes allow for efficient water flows, reducing total demands. It could also indicate less resistance to flow, requiring less pressure to move the same quantities of water and experiencing lower friction losses, where less water would be lost due to the internal resistance of the pipes.

**Figure 9:**
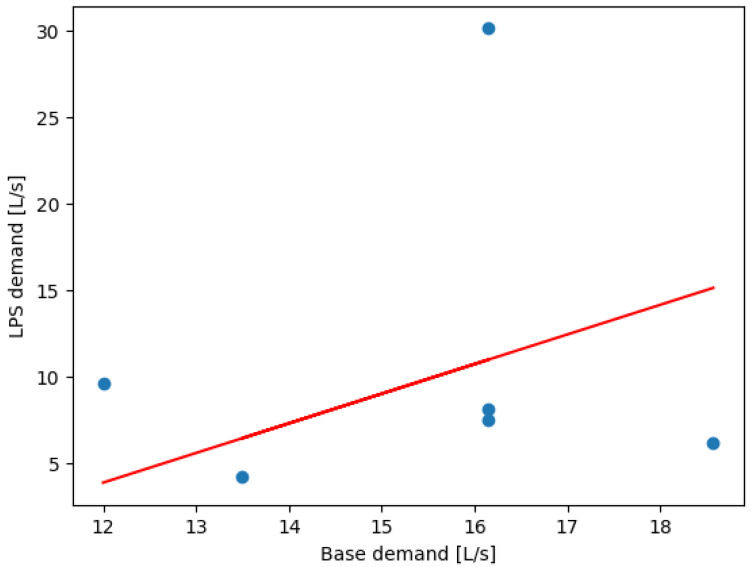
Linear regression between $B_{D}$ and $L_{D}$ in Moratalla.

A base demand of 16.15 L/s was calculated, oscillating between [13.72, 18.57]. The simulation results show sufficient flow for most nodes. Linear regression (Figure 9) predicts node demands based on base demands.

Unlike Lugo, Moratalla shows a negative correlation between base demand and pipe diameter. This result indicates that smaller pipes are sufficient to meet demand in areas with smaller populations and less complex water networks, while larger pipes may be underutilized. The negative slope (−0.0200) highlights that as pipe diameter increases, the base demand slightly decreases, potentially due to water distribution across a broader network with less intense demand at individual nodes. In fact, 70.3% of nodes were optimized, achieving an MAE of 7. The linear regression between base demand and pipe diameter (Figure 9) shows a negative slope, indicating lower base demands for larger diameters.

(4) $$B_{D}=-0.02003613\times \mathrm{diameter}+18.60030081$$

Moratalla’s extensive water supply network is largely due to its geographic layout. It serves a dispersed rural population and agricultural areas, contrasting with more concentrated urban populations in the two other cities.

**Figure 10:**
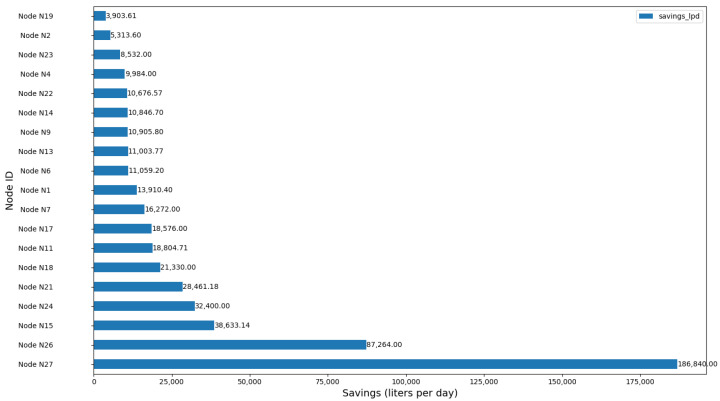
Resource savings per node optimized in Moratalla.

Figure 10 shows resource savings per node, with the highest saving node achieving 186,840 L/day.

### 5.3. Balerma Scenario

**Figure 11:**
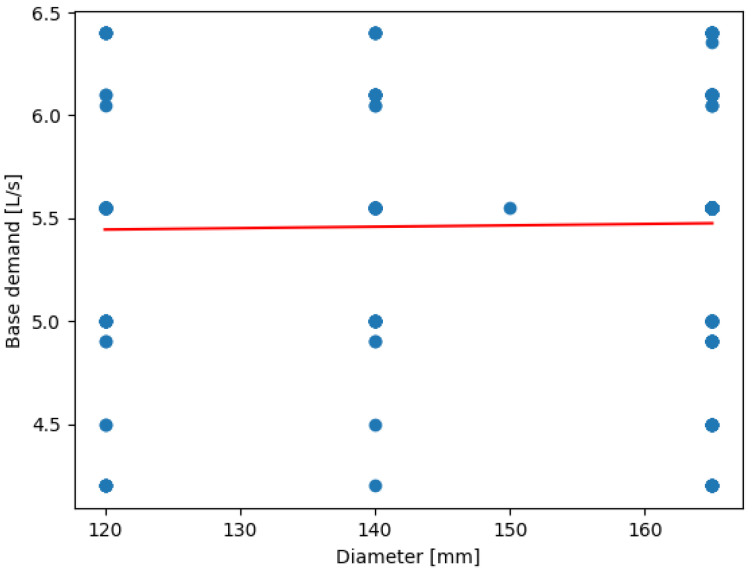
Linear regression between $B_{D}$ and pipe diameter at Balerma.

Figure 11 displays the performed linear regression between the base demand and pipe diameter. Varying the pipe diameter between 120, 140, and 165 mm, it is found that the base demand tends to stabilize at a single value for any one diameter, establishing that base demand=k, implying that pipe diameter does not have a significant impact on the base demand in the range of values considered.

**Figure 12:**
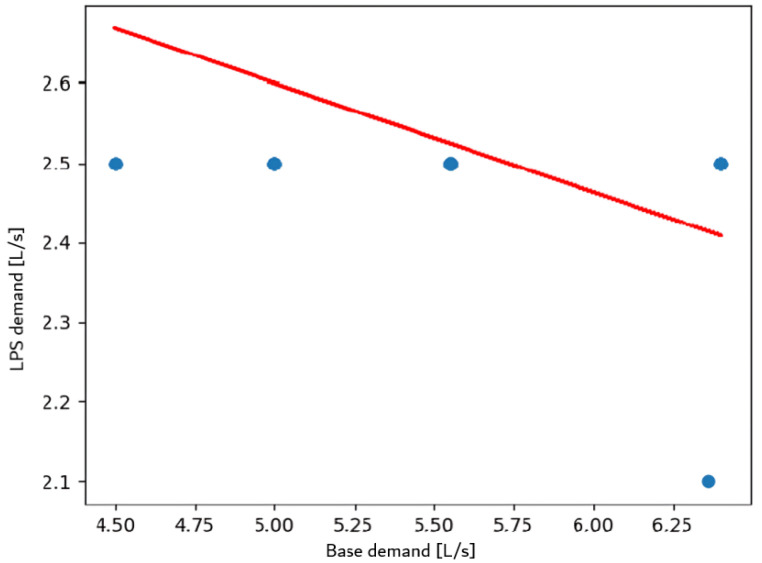
Linear regression between $B_{D}$ and $L_{D}$ in Balerma.

A base demand of 5.53 L/s was calculated, oscillating between [4.7, 6.35]. The linear regression between base demand and lps demand (Figure 12) shows a negative trend. Of 445 nodes, 430 were optimized, achieving an MAE of 0.049.

The stabilization of base demand across varying pipe diameters in Balerma reflects the relatively uniform demand patterns within its water distribution network. This is likely due to the town’s smaller size and more consistent water usage across its nodes. The flat slope (0.0007) indicates a minimal change in base demand with increasing diameter, suggesting that pipe size is less critical in this scenario than in other cities. This stability is ideal for predictive modeling as it simplifies resource planning and optimization efforts. The distribution of resource savings in Balerma reveals that certain nodes, particularly those with higher local demand, offer greater opportunities for water conservation. The top-saving node (186,840 L/day) demonstrates the effectiveness of the optimization strategy in reducing water wastage. This trend suggests that optimizing nodes in high-demand areas disproportionately positively impacts overall network efficiency. Additionally, the range in savings across nodes reflects the heterogeneity of water usage within Balerma’s network, which is influenced by the varied topography and population density. The linear regression between base demand and pipe diameter (Figure 12) shows stabilization of base demand values across diameters.

(5) $$B_{D}=0.0006776\times \mathrm{diameter}+5.3639962$$

**Figure 13:**
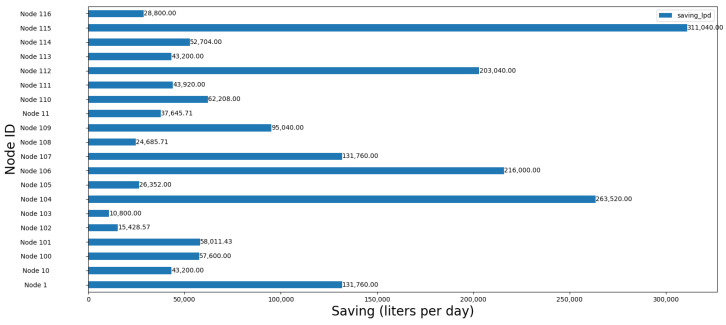
Resource savings per node optimized in Balerma.

Figure 13 shows resource savings per node, respectively, with the highest saving node achieving 311,040 L/day.

In Balerma, the highest resource-saving node saves 311,040 L/day, indicating that substantial water savings can be realized through targeted optimization even in smaller cities. The relatively high number of optimized nodes (430) reflects the potential for widespread efficiency improvements across the network. The consistency in node savings indicates a more homogeneous distribution of water demand, likely due to the town’s uniform population density and lower overall water consumption compared to larger urban areas. Table 3 presents the results obtained from Algorithm 1, showing key performance indicators for the three cities Lugo, Moratalla, and Balerma.

This table highlights the performance of the optimization algorithm across the three cities—Lugo, Moratalla, and Balerma—based on key metrics such as mean absolute error (MAE), the number of optimized nodes, and total resources optimized. The differences in MAE values across the cities are likely due to the unique characteristics of their water distribution networks. Balerma, with a smaller, less complex network and more uniform water demand patterns, allows for more accurate predictions. The near-perfect MAE of 0.05 suggests that the optimization algorithm can capture demand variations with minimal error in such environments. Conversely, Lugo has a more complex network, higher population density, and greater variability in demand between nodes. This complexity likely contributes to the higher MAE (46.10), as the algorithm struggles to predict demand in a more dynamic system accurately. Future iterations of the algorithm could include more advanced modeling techniques, such as clustering similar demand nodes, to reduce error in complex systems like Lugo. This could be attributed to the city’s larger and more complex water network, which introduces greater variability in local demand. Despite the lower prediction accuracy, Lugo has the highest resource optimization potential, optimizing over 76 L per second. Moratalla, with a moderate MAE (7.08), falls between the other two cities regarding prediction accuracy and resource optimization. The relatively lower total optimized resources (7.27 L/s) reflect its smaller water distribution network and population size. Overall, the table underscores the importance of tuning the optimization algorithm to the specific characteristics of each city’s water network for maximum effectiveness.

In Table 4, a comparative analysis of resource savings demonstrates the varying levels of efficiency achieved across the different cities regarding water resource management. This table highlights how the optimization strategies applied to each city resulted in differing amounts of water savings.

This table presents the results of the linear regression model used to predict base demand based on pipe diameter in the three cities. The regression coefficients and intercepts for Lugo, Moratalla, and Balerma reveal distinct trends in how base demand scales with pipe diameter in each city’s water network. The positive regression coefficient in Lugo (0.1847) suggests that as pipe diameters increase, so does base demand. This is logical for a large urban center like Lugo, where population density and infrastructure requirements necessitate larger pipes to meet higher demand. In contrast, Moratalla’s negative coefficient (−0.0200) indicates that larger pipes are less frequently used or underutilized in some areas, likely due to lower population density or water distribution across a more spread-out network. This negative relationship may also point to inefficiencies in pipe allocation in Moratalla, where pipe size does not match actual demand patterns, signaling a need for infrastructure adjustments to optimize water flow better. This indicates a more dispersed network with varying demand patterns, where larger pipes might be underutilized in certain areas. Balerma’s near-zero regression coefficient (0.0007) reflects the stability of base demand across different pipe diameters, characteristic of its smaller, more homogenous network. The relatively small variations in demand make it easier to predict resource needs accurately. The intercept values further highlight the differences between the cities, with Lugo showing the highest intercept (119.06 L/s), representing the base demand in areas with high water usage. In comparison, Balerma’s low intercept (5.36 L/s) reflects its smaller scale. This analysis demonstrates that network topology, population distribution, and infrastructure complexity significantly influence the relationship between pipe diameter and base demand, offering insights for more tailored optimization strategies. The data reveal that cities with more optimized nodes, such as Balerma, achieved more significant savings due to their smaller population. In contrast, cities like Lugo and Moratalla experienced a more moderate improvement. This comparative approach allows for a better understanding of how localized factors—such as network size, population density, and infrastructure complexity—affect the effectiveness of resource-saving algorithms. Additionally, it emphasizes the importance of tailoring optimization strategies to the specific characteristics of each water distribution system to maximize efficiency and sustainability.

### 5.4. Summary of Results

The results obtained in this study were analyzed for water distribution networks in Lugo, Moratalla, and Balerma using EPANET simulations and linear regression techniques. The results show a substantial difference in the MAE between Lugo (46.10) and Balerma (0.05), which can be attributed to Lugo’s larger and more complex network. Additionally, Balerma’s high number of optimized nodes (430 out of 445) reflects its network’s ability to achieve significant resource efficiency despite its smaller population. These findings suggest that optimization efforts should be concentrated in areas with high node density for maximum efficiency gains. We highlight the following points:

1. Demand Prediction Accuracy: The study highlighted the variability in demand prediction accuracy across different scenarios. Balerma achieved the highest accuracy with an MAE of 0.049, next was Moratalla with an MAE of 7.08, and finally, Lugo had the lowest accuracy (MAE of 46.10).
2. Resource Optimization: Resource optimization varied significantly, with Balerma optimizing 430 out of 445 nodes, Moratalla 19 out of 27 nodes, and Lugo 10 out of 28 nodes. The highest resource savings per node were observed in Lugo and Balerma.
3. Relationship Between Base Demand and Pipe Diameter: The linear regression analyses showed distinct trends. Lugo displayed a positive correlation between base demand and diameter, while Moratalla showed a negative correlation. Balerma’s base demand stabilized across different diameters, indicating a minimal diameter impact on base demand.

While the simulation results have highlighted the significant potential for optimization in urban water networks, the next logical step is to translate these findings into a practical solution. To ensure real-world applicability, we propose a scalable, technology-driven architecture that builds on these insights, enabling optimized water distribution systems deployment across diverse urban environments. This architecture leverages sensors, wireless communication, and cloud-based data processing to provide real-time monitoring, anomaly detection, and predictive modeling, ensuring effective and sustainable water management. It features sensors in nodes, LoRaWAN, and satellites for global wireless communication and cloud-based data processing for real-time monitoring, anomaly detection, and predictive modeling.

**Figure 14:**
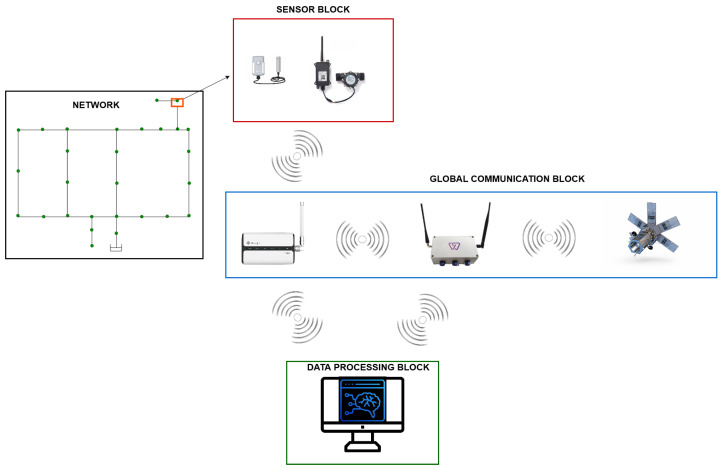
General system architecture.

While the proposed architecture offers a promising blueprint for optimizing water distribution systems, practical challenges must be addressed to ensure real-world applicability. These include the integration costs of deploying IoT sensors across vast urban networks, ensuring the accuracy and reliability of real-time sensor data, and managing large-scale data processing requirements. Additionally, securing reliable wireless communication (e.g., LoRaWAN) in urban areas with potential signal interference or remote areas with less coverage could pose challenges. Overcoming these obstacles will require collaborations with local water authorities, pilot projects in urban centers, and further refinement of the system architecture based on real-world feedback, as seen in Figure 14.

The sensor block, comprising water level and water flow sensors, will be strategically installed at reservoir nodes to obtain real-time measurements. Water flow sensors will detect anomalies like leaks, triggering alarms to alert users. Utilizing LoRaWAN wireless technology, sensor readings will be transmitted to a satellite or data-processing center. A LoRaWAN gateway will facilitate communication between the sensors and a constellation of LEO satellites, enhancing the global availability of water level data for expedited decision-making in resource optimization. Data received by the gateway will be forwarded to a cloud-based server for storage, ensuring scalability, performance, and sensor range across multiple device platforms. Additional sensors like soil moisture sensors may be integrated to enable predictive modeling and facilitate real-time decision-making based on environmental conditions. For example, soil moisture sensors would complement the system by allowing predictive models when the sensors detect a decrease in the humidity percentage, expediting decision-making regarding the real-time environment.

The proposed architecture addresses the technical challenges of urban water distribution and supports sustainable resource management by enabling precise control over water networks. Its flexible, modular design suits diverse urban environments, from small towns like Balerma to larger, more complex cities like Lugo. Integrating IoT, satellite communication, and cloud computing forms the foundation for smarter, more responsive water distribution systems capable of adapting to changing demand and environmental conditions.

Our results demonstrate significant improvements in water conservation compared to traditional water distribution models, largely due to the integration of predictive AI and real-time IoT data. Unlike prior studies limited to static optimizations, our approach dynamically adjusts to fluctuating demand, achieving up to a 96.62% reduction in wastage. However, our methodology has limitations. Firstly, scalability remains challenging; the model may require adaptation to handle more extensive urban networks or datasets. Secondly, while EPANET suits our simulations, a real-world implementation may encounter data limitations or connectivity constraints. Lastly, model adaptability is affected by urban infrastructure variances, and additional customization may be necessary in cities with older or highly complex water networks.

After analyzing the results, we also determined significant challenges in implementing this technology in urban environments with outdated infrastructure. From our experience and expertise, we observed that these challenges often include limitations in energy capacity, deficiencies in network connectivity, and difficulties in adapting legacy systems to modern technologies. Consequently, we identified that achieving successful and sustainable integration requires implementing solutions that minimize additional infrastructure needs and maximize energy efficiency. These findings lead us to propose strategies and approaches that address these challenges through modular technology use, energy feedback options, and close collaboration between the public and private sectors.

## 6. Conclusions and Future Work

This study demonstrates the potential of AI and IoT in optimizing urban water networks, achieving significant water savings with a 96.62% optimization rate. Although it is apparent that nodes with fewer inhabitants have lower water consumption, the more crucial insight is the impact of distribution efficiency, leak minimization, and network optimization on overall resource savings, even for smaller populations. The following key contributions were derived from the conclusions:

1. Methodological Framework: the integration of EPANET simulations with linear regression techniques provides a robust framework for analyzing and optimizing water distribution networks.
2. Scenario-Based Analysis: the study’s scenario-based approach offers valuable insights into different urban water distribution networks’ unique characteristics and optimization potentials.
3. Optimization Insights: the results provide actionable insights for water resource managers, highlighting nodes with significant optimization potential and contributing to more efficient water distribution system designs.
4. Predictive Modeling: the developed predictive models can be utilized for future planning and management, assisting in making data-driven decisions for infrastructure improvements.

Overall, this research contributes to understanding water distribution dynamics and applying predictive analytics in enhancing water resource management.

This study has several practical implications for urban water management and public policy. The proposed system offers an economically viable means of reducing operational costs by minimizing water wastage and enhancing resource allocation through predictive insights. Maintenance strategies can leverage real-time data for timely interventions, reducing downtime and extending infrastructure lifespan. Additionally, adopting this AI- and IoT-based architecture can assist urban policymakers in addressing water scarcity and resilience goals. For effective integration, local governments might consider phased rollouts that prioritize high-demand areas, align with existing infrastructure, and promote collaboration between public utilities and technology providers to ensure sustainable implementation. Future research will focus on conducting real-world pilot studies in collaboration with local water authorities to validate the scalability of the proposed architecture in practical settings, where it can be addressed the integration of EPANET with real-time data collection systems and upgrading tools capable of handling larger networks, such as coupling EPANET with Python-based optimization for the improvement of modeling precision. These pilots will explore the feasibility of deploying and integrating sensors into existing infrastructure, focusing on cities facing varying environmental challenges. Additionally, we plan to further develop advanced AI techniques, such as deep learning or graph-based models, to enhance prediction accuracy, particularly in more complex networks like Lugo. Exploring the potential for integrating water systems with other urban services, such as energy or waste management, will also be a key research avenue. Such integration could lead to comprehensive smart city solutions, contributing to developing more sustainable and resilient urban environments. Additionally, there are plans to deploy sensors within real-world water distribution infrastructure, enabling the validation of simulation results with real-time data to fine-tune optimization models.

Moreover, we plan to explore the impact of incorporating dynamic unit_cost values, perhaps linked to environmental data or predictive trends. This adjustment could improve the model’s adaptability and responsiveness to changing conditions, further aligning our optimization goals with sustainable water resource management. In addition, advanced artificial intelligence techniques, such as deep learning or graphical neural networks joined with evolutionary optimization algorithms, will be explored further to enhance the efficiency and accuracy of the proposed solutions. These techniques will allow for more effective optimization in dynamic scenarios, adapting to fluctuating supply and demand conditions. Further research will also investigate integrating water distribution systems with other urban services, such as energy and transportation networks, advancing towards developing sustainable smart cities.

Finally, collaborations with local authorities and international organizations will be sought to bring these solutions to a practical level, implementing large-scale pilot projects in cities with diverse climate and geographic conditions beyond the Mediterranean-type climate already evaluated.

Therefore, with this work for the future, we aim to provide a foundation for addressing climate challenges through institutional efforts while also helping to secure funding by highlighting the critical importance of water network management in large cities and raising awareness about the issue of drought.

## Figures and Tables

**Table 1:** Comparison of Our Proposal with Related Works.

Work	Water Anal.	AI	Opt.	Tech.	Sust.	Arch.
Sahu et al. [10]	🗸		🗸	🗸	🗸	
Glenndon et al. [11]				🗸	🗸	🗸
Bharucha et al. [12]	🗸		🗸	🗸	🗸	🗸
Serafeim et al. [13]			🗸	🗸	🗸	
Sakomoto et al. [14]	🗸		🗸	🗸	🗸	
Ramos et al. [15]		🗸	🗸	🗸	🗸	🗸
ChiaCheng et al. [16]			🗸	🗸		🗸
Ajaz et al. [17]	🗸		🗸	🗸		
Mabrok et al. [18]	🗸		🗸	🗸	🗸	🗸
Bilal et al. [19]			🗸			
Martínez et al. [20]				🗸	🗸	🗸
Kinman et al. [21]			🗸	🗸		🗸
Zeng et al. [22]	🗸	🗸		🗸		🗸
Nova et al. [23]	🗸	🗸			🗸	
Villar et al. [24]	🗸	🗸		🗸	🗸	🗸
Lewis et al. [25]	🗸			🗸		🗸
Kanaga et al. [26]		🗸		🗸		🗸
Henderi et al. [27]			🗸	🗸	🗸	
Our proposal	🗸	🗸	🗸	🗸	🗸	🗸

**Table 2:** Inhabitants (inhab.) per Node for Lugo, Moratalla, and Balerma.

Node	1	2	3	4	5	6	7	8	9	10	11	12	13	14	15	16	17	18	19	20	21	22	23	24	25	26	27	28
**#inhab. Lugo**	50	60	100	70	35	41	13	74	13	86	24	25	44	10	9	5	11	13	16	17	20	90	88	73	68	50	98	8
**#inhab. Moratalla**	50	100	140	90	67	75	42	31	98	86	67	23	52	73	35	62	20	47	83	90	17	70	120	10	31	5	4	6
**Node for Balerma**	1	10	100	101	102	103	104	105	106	107	108	109	11	110	111	112	113	114	115	116								
**#inhab. Balerma**	2	5	3	7	14	20	1	10	1	2	7	4	7	5	6	2	4	5	1	6								

**Table 3:** Results of Algorithm 1.

Parameter	Lugo	Moratalla	Balerma
MAE	46.10	7.08	0.05
Optimized nodes	10/28	19/27	430/445
Total optimized resources	76.45	7.27	2.88

**Table 4:** Results of Algorithm 2.

Parameter	Lugo	Moratalla	Balerma
Regression coefficient	0.1847	−0.0200	0.0007
Intersection	119.06	18.60	5.36

## Data Availability

The datasets used and/or analyzed during the current study are available from the corresponding author upon reasonable request.

## References

[1] Moreno Sanz F. (1995). El agua en España: Un recurso aforado para un consumo desaforado. Razón Y Fe.

[2] Rico Amorós A.M. (2004). Sequías y abastecimientos de agua potable en España. Boletín Asoc. Geógrafos Españoles.

[3] Martínez Gil F.J. (2007). Los problemas del agua en España: Análisis de una realidad. Enseñanza Cienc. Tierra Rev. Asoc. Española Para Enseñanza Cienc. Tierra.

[4] Seral M.A.C. (2015). Aplicaciones basadas en tecnologías de la información geográfica para ayudar a gestionar el agua de riego en comunidades de regantes. XXIV Congreso de la Asociación de Geógrafos Españoles. Análisis Espacial y Representación Geográfica: Innovación y Aplicación.

[5] Fedra D.K., Austria G. (2005). Water Resources Simulation and Optimization: A Web Based Approach.

[6] Parada R., Font J., Casas-Roma J. (2019). Predicting Energy Generation Using Forecasting Techniques in Catalan Reservoirs. Energies.

[7] Parada R., Font J., Casas-Roma J., Torra V., Narukawa Y., Pasi G., Viviani M. (2019). Forecasting Water Levels of Catalan Reservoirs. Modeling Decisions for Artificial Intelligence.

[8] Tarazona Lizarraga C. (2020). Análisis de las necesidades de una Smart City en el marco de un desarrollo sostenible. Master’s Dissertation.

[9] Agency U.E.P. (2020). EPANET2.2. https://github.com/USEPA/EPANET2.2/releases/tag/2.2.0.

[10] Sahu B., Singh A. (2020). Optimal Design and Analysis of Water Distribution Networking Systemusing EPANET. J. Water Eng. Manag..

[11] Boniel G.J.M., Catarinen C.C., Nanong R.D.O., Noval J.P.C., Labrador C.J.M. (2020). Water management system through wireless sensor network with mobile application. AIP Conf. Proc..

[12] Bharucha A., Maheshwari A., Gudi R.D. An EPANET—MATLAB Framework for Quality and Quantity Management in Intermittent Water Supply Network. Proceedings of the 2022 Eighth Indian Control Conference (ICC).

[13] Serafeim A.V., Perdios A., Fourniotis N.T., Langousis A. (2023). Towards More Efficient Hydraulic Modeling of Water Distribution Networks Using the EPANET Software Engine. Environ. Sci. Proc..

[14] Sakomoto T., Lutaaya M., Abraham E. (2020). Managing Water Quality in Intermittent Supply Systems: The Case of Mukono Town, Uganda. Water.

[15] Ramos H.M., Kuriqi A., Besharat M., Creaco E., Tasca E., Coronado-Hernández O.E., Pienika R., Iglesias-Rey P. (2023). Smart Water Grids and Digital Twin for the Management of System Efficiency in Water Distribution Networks. Water.

[16] Chia-Cheng S. (2024). Enhancing the EPANET Hydraulic Model through Genetic Algorithm Optimization of Pipe Roughness Coefficients. In Water Resour Manage..

[17] Ajaz M., Ahmad D. (2023). Optimal Water Quality Simulation of the Proposed Water Distribution System for the University of Kashmir Using EPANET 2.2 and Leakage Modelling of the Network Using EPANET Extension-WaterNetGen. Environ. Sci. Proc..

[18] Mabrok M.A., Saad A., Ahmed T., Alsayab H. (2022). Modeling and simulations of Water Network Distribution to Assess Water Quality: Kuwait as a case study. Alex. Eng. J..

[19] Mirza B., Pant M., Snasel V. (2021). Design Optimization of Water Distribution Networks through a Novel Differential Evolution. IEEE Access.

[20] Moreno M.M., Castrejón D.A.H., Honorato J.C., Santamarina M.L.G., Moreno B.G. (2023). Water network: Aplicación móvil con tecnología Arduino para la medición de presión y consumo del agua. Cienc. Lat. Rev. Científica Multidiscip..

[21] Kinman G., Žilić Ž., Purnell D. (2023). Scheduling Sparse LEO Satellite Transmissions for Remote Water Level Monitoring. Sensors.

[22] Zeng H., Dhiman G., Sharma A., Sharma A., Tselykh A. (2023). An IoT and Blockchain-based approach for the smart water management system in agriculture. Expert Syst..

[23] Nova K. (2023). AI-Enabled Water Management Systems: An Analysis of System Components and Interdependencies for Water Conservation. Eig. Rev. Sci. Technol..

[24] Villar Miguelez C., Monzon Baeza V., Parada R., Monzo C. (2023). Guidelines for Renewal and Securitization of a Critical Infrastructure Based on IoT Networks. Smart Cities.

[25] Lewis G., Snider B., Vamvakeridou-Lyroudia L., Chen A.S., Djordjević S., Savić D.A. (2023). Web-based GIS for simulation, and visualisation: EPANET and data-rich shapefiles. IOP Conf. Ser. Earth Environ. Sci..

[26] Kanaga Suba Raja S., Usha Kiruthika S., Raman C.J. A Novel Water Distribution and Performance Monitoring System using AI. Proceedings of the 2024 8th International Artificial Intelligence and Data Processing Symposium (IDAP).

[27] Henderi H., Sejati W., Pranata S., Yusup M., Hardini M., Yusuf N.A. Innovative Approaches in Smart Hydrological Monitoring for Urban Water Resource Sustainability. Proceedings of the 2024 3rd International Conference on Creative Communication and Innovative Technology (ICCIT).

[28] Bi W., Dandy G., Maier H. (2015). Improved genetic algorithm optimization of water distribution system design by incorporating domain knowledge. Environ. Model. Softw..

